# Defects in London Methods

**Published:** 1921-02-12

**Authors:** 


### A TUBERCULOSIS DISPENSARY SURVEY.

### Defects in London Methods.

A SURVEY just made by the Ministry of Health relative to the organisation in London of schemes for the dispensary treatment of tuberculosis has revealed certain deficiencies, for which remedies are now suggested by the Ministry for the consideration of the various borough councils.

The survey showed, amongst other matters, that the importance of arriving at a definite diagnosis as quickly as possible has not in all cases been sufficiently appreciated, and that the facilities provided at the appointed consultative hospitals have not been fully utilised; that the provision of routine treatment, as distinct from consultative work, has at some dispensaries been developed to an undesirable extent, particularly by the giving of medicines, drugs, etc., to patients for prolonged periods; that such necessary functions of the dispensaries as home visiting, the examination of contacts of newly notified cases, and the following up of patients ceasing to attend the dispensaries, have not been adequately developed in many instances; and that, generally, the work of dispensary staffs has not been sufficiently co-ordinated with the preventive work of the public health departments of the borough councils. Further, it is necessary that the days and hours of opening and the specific purposes of the dispensary sessions should be arranged in accordance with definite objects—namely, the effective organisation of the work of the dispensaries, the convenience of different classes of patients resorting to them, and the avoidance of long periods of waiting by patients.

### The Ministry's Suggestions.

Subjoined are the suggestions which the Ministry now puts forward with a view to securing that the essential functions of the dispensary service—in relation both to the prevention and treatment of tuberculosis—are adequately fulfilled:

1. Arrangements should be made for the complete co-ordination of the work of the dispensary with that of the public health department, and if this is to be secured it will be necessary for each tuberculosis officer to be appointed as an assistant to the M.O.H. for tuberculosis work, and be given a responsible share of the control under the administrative direction of the M.O.H. of the whole of the tuberculosis work in the portion of the borough served by his dispensary.

2. The working week of a whole-time tuberculosis officer should not in any case be less than thirty-six hours, and it is suggested that, as a normal basis, there should be one whole-time tuberculosis officer to about every 160 deaths from tuberculosis per annum. In dispensary areas which require on this basis the services of more than one whole-time tuberculosis officer, but less than two whole-time officers, part-time assistant tuberculosis officers might be appointed; and there should be at least one dispensary nurse, whole- or part-time, for each whole-time tuberculosis officer or part-time assistant tuberculosis officer. These nurses should work under the direct control of the tuberculosis officer, and their work should be properly co-ordinated with the general work of the dispensary.

In those cases in which the adoption of the suggested basis would involve an addition to the existing staff of the dispensary, the borough council will, of course, have regard to the present financial position of the borough.

3. The Ministry understands that the London County Council proposes to arrange for the provision of a sufficient number of consulting centres to serve the whole of the dispensaries in London. These consulting centres, equipped with observation beds and having physicians with special experience in tuberculosis, will be provided, if possible, at certain of the London hospitals. Tuberculosis officers will have access to the observation beds, and consultations upon the occupants of these beds and upon other patients brought by tuberculosis officers should take place at regular intervals. When this provision has been made, the existing arrangements of the borough councils with hospitals will be unnecessary, as each dispensary will be linked to one of the consulting centres.

It is anticipated that these consulting centres will prove of considerable assistance to tuberculosis officers in their work. The provision of a large number of observation beds should be the means of ensuring that each case receives the appropriate form of treatment, whether residential or otherwise, and should improve and cheapen the working of the tuberculosis scheme in London

4. In order to arrive at the earliest possible date at a definite diagnosis in doubtful cases of tuberculosis which present themselves at the dispensaries, there should be shorter periods of observation combined with more intensive study of each case, full use being made of the facilities and aids to diagnosis afforded by the Consulting Centre.

5. Treatment at the dispensary, as distinct from diagnosis, consultation, and general supervision, should as a rule be limited to patients whose continued treatment requires special knowledge or technical skill, and to those who are unable to obtain other adequate medical attendance. Patients who require treatment which can, consistently with the best interests of the patient, be properly undertaken by a general practitioner of ordinary professional competence and skill, and who are either insured persons or can afford to pay for medical attendance, should not be encouraged to attend the dispensary for routine treatment.

6. The practice of treating patients at the dispensaries on a large scale and over prolonged periods with bottles of medicine, cod-liver oil, etc., and of giving medicines to ensure the attendance of patients, should be discouraged. Patients should rather be educated out of the belief in the efficacy of drugs, and be taught the value of personal advice and of instruction in a hygienic mode of life.

Further recommendations deal with the desirability of full co-operation between the dispensary service and the school medical service and the means to secure it; preventing patients being kept waiting at a dispensary; the examination of "contacts" and then following up of patients; co-operation between the tuberculosis officer and local medical practitioners; provision of the necessary clerical assistance.

In inviting consideration of the foregoing suggestions, the Ministry recalls the Order of the National Health Insurance Joint Committee stipulating that sanatorium benefit would cease to be included in the benefits conferred on insured persons on January 1. It has been necessary to make further use of the powers under that Order to fix May 1 as the date of discontinuance of sanatorium benefit, which therefore will continue to be administered by Insurance Committees till that date.

